# Recombination Phenotypes of *Escherichia coli greA *Mutants

**DOI:** 10.1186/1471-2199-12-12

**Published:** 2011-03-31

**Authors:** Anthony R Poteete

**Affiliations:** 1 Department of Microbiology and Physiological Systems, University of Massachusetts Medical School, Worcester, MA, USA

## Abstract

**Background:**

The elongation factor GreA binds to RNA polymerase and modulates transcriptional pausing. Some recent research suggests that the primary role of GreA may not be to regulate gene expression, but rather, to promote the progression of replication forks which collide with RNA polymerase, and which might otherwise collapse. Replication fork collapse is known to generate dsDNA breaks, which can be recombinogenic. It follows that GreA malfunction could have consequences affecting homologous recombination.

**Results:**

*Escherichia coli *mutants bearing substitutions of the active site acidic residues of the transcription elongation factor GreA, D41N and E44K, were isolated as suppressors of growth inhibition by a toxic variant of the bacteriophage lambda Red-beta recombination protein. These mutants, as well as a D41A *greA *mutant and a *greA *deletion, were tested for proficiency in recombination events. The mutations were found to increase the efficiency of RecA-RecBCD-mediated and RecA-Red-mediated recombination, which are replication-independent, and to decrease the efficiency of replication-dependent Red-mediated recombination.

**Conclusion:**

These observations provide new evidence for a role of GreA in resolving conflicts between replication and transcription.

## Background

The GreA protein is an evolutionarily-conserved bacterial transcriptional factor, which interacts directly with RNA polymerase. Binding of GreA to paused and backtracked transcriptional complexes induces RNA polymerase to hydrolyze the last few nucleotide residues at the 3' end of the nascent transcript protruding beyond the enzyme's catalytic center, and resume transcription, in vitro [1] and in vivo [2,3]. The extensively characterized interaction between RNA polymerase and GreA leads most simply to the idea that GreA functions in transcriptional regulation. Consistent with this idea, *E. coli *lacking GreA protein exhibits altered levels of expression of some of its genes [4,5], as well as of the transcript encoding bacteriophage λ proteins which are synthesized late in infection [6]. However, the magnitude of the gene expression effects is small, and a clear understanding of GreA's biological function remains to be obtained. Some recent studies have provided evidence that GreA may have a role in resolving conflicts between replication and transcription [7,8].

In this study, *greA *mutants are shown to have effects on homologous recombination which can be most readily explained by a replication fork-preserving function of wild type GreA. These mutants were isolated in a screen for host functions which affect the cell's interaction with the Redβ recombination protein of bacteriophage λ.

In wild-type *E. coli*, homologous recombination depends primarily on the function of two proteins, the RecA synaptase and the RecBCD helicase/nuclease. For genetic engineering purposes, RecBCD is often replaced by the Red recombination system of bacteriophage λ [9]. The Red system, consisting of the single-strand annealing protein Redβ and the exonuclease λExo, can substitute for RecBCD in promoting RecA-dependent homologous recombination [10,11]. In addition, however, Red confers new capabilities on the cell, including the ability of the cell's chromosome to recombine with short linear DNA species, even in the absence of RecA [12,13]. In this RecA-independent mode of recombination, Red apparently acts by targeting the replisome, leading to either strand assimilation or a template switch [14,15].

## Results

To probe interactions between the Red system and cellular proteins, Redβ was fused at its C-terminus to the SPA affinity tag [16] on a medium copy number plasmid, under control of the *lac *repressor. Redβ-SPA was found to be toxic, inhibiting the growth of wild type *E. coli *when induced. This toxicity is surprising, because Redβ itself is innocuous, and hundreds of non-toxic SPA-tagged *E. coli *proteins have been made and characterized [17]. One possible explanation is that the synthetic toxicity of Redβ-SPA results from the delivery of the SPA tag to a critical site, possibly the normal site of Redβ action in the cell. As a hypothetical example, if Redβ normally binds to a replisome protein, it would necessarily have evolved to do so without interfering with replication. However, Redβ with the 69 amino acid residue SPA tag appended might bind to its normal site and clash sterically with another component of the replisome. According to this reasoning, cellular mutants altered in their interaction with Redβ could be selected as suppressors of Redβ-SPA toxicity. Several such suppressor mutants were isolated. Initial mapping showed that three of the mutations mapped to the same area in the *E. coli *chromosome. Fine-structure mapping and sequencing revealed that these three mutants each encoded a GreA protein with a single amino acid substitution: D41N (isolated twice), or E44K. The affected residues are particularly significant in GreA's activity. Both participate in the coordination of Mg^++ ^ions in the catalytic core of RNA polymerase [18-20]. The *greA*-D41N mutation was found not to affect the level of either Redβ-SPA or Redβ as detected by western blot (not shown), ruling out the simplest explanation--that the *greA *mutants suppress the toxicity of Redβ-SPA by preventing its synthesis.

To study the unexpected relationship between GreA and Red, *E. coli *strains were constructed bearing the *greA *D41N and E44K mutations, as well as a D41A mutation and a simple *greA *deletion, in a variety of genetic backgrounds. The strains were found to have a number of recombination-related phenotypes.

Consistent with the idea that the *greA *mutants might be affected in their interaction with Red, λ behaves as if it had a mutation in either *red *gene (*bet *or *exo*), or both, when infecting these mutant bacteria. A λ *red gam *double mutant does not form plaques on a *recA *host, though *red *and *gam *single mutants do so [21]. As indicated in Table 1, a λ *gam *single mutant fails to form plaques on a *recA *host when the *recA *host additionally bears a *greA *point mutation (but not a *greA *deletion). A deletion eliminating both *red *genes reduces λ plaque size; similarly, the *greA *point mutations in the host reduce the size of wild type λ plaques. As might be expected if mutating either *red *or *greA *is equivalent in this test, the *greA *point mutations have less effect on the plaque size of a λ *red *mutant than on the plaque size of λ wild type. This latter observation suggests that the *greA *point mutation primarily affects λ *red *function, rather than λ transcription, but does not rule out the possibility that there might be some effect on transcription.

**Table 1:** λ plaque formation^a^

Host genotype^b^	λ wild	λ *gam*	λ *red*
wild type	++	+	+

*greA-D41N*	+	-	+

*greA-D41A*	+	-	+

*greA-E44K*	+	-	+

*greAΔ*	++	+	+

*greBΔ*	++	+	+

*greBΔ greA-D41N*	+	-	+

a. ++ large plaques, + small plaques, - no plaques

b. All strains bear a *recAΔtet *substitution.

**Figure 1:**
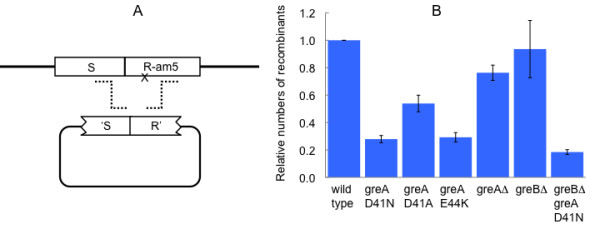
**Marker rescue**. A. λ *cI857 R-am5*, bearing an amber mutation in the endolysin gene R, can form a plaque on the non-suppressing host only if it recombines with the plasmid or reverts. B. Efficiencies of mutants relative to wild type. Titers of the phage on cells bearing the homology-containing plasmid, minus titers on cells bearing the vector (revertants) are determined, and divided by the wild type value. Means and standard errors of four experiments are shown. All strains are *recA*.

The effects of *greA *mutants on λ recombination were tested more directly in a marker rescue test, shown in Figure 1. The crosses were done in a *recA *host, to restrict recombination to the Red-promoted, replisome invasion pathway [15]. Marker rescue was reduced 3-4 fold by the D41N and E44K substitutions, 2-fold by D41A, and very slightly by the *greA *deletion. The effects of the *greA *mutations on Red-mediated recombination are modest compared to the effects of a *red *deletion mutant, which reduces plaque formation in this test to the background of reversion, approximately 40-fold (data not shown).

**Figure 2:**
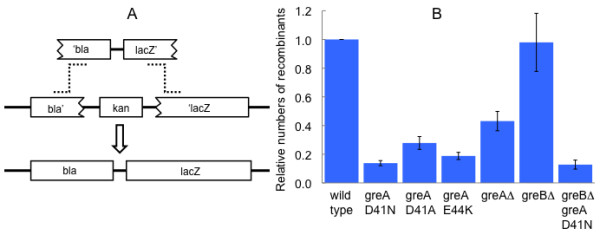
**Red-mediated chromosomal gene replacement**. A. Strains bearing an alteration of the *lac *locus in which *lacI *through the N-terminal coding region of *lacZ *is replaced by the C-terminal two-thirds of the *bla *gene and the *aph *gene of Tn903 can recombine with a linear dsDNA generated by PCR and introduced by electroporation, creating an ampicillin-resistant, Lac+ recombinant. B. Efficiencies of mutant strains relative to wild type. Means and standard errors of three or four experiments are shown.

The *greA *mutations also affect Red-mediated recombination outside the context of a phage infection. As shown in Figure 2, the efficiency of recombination between the bacterial chromosome and a linear dsDNA introduced by electroporation is reduced in the *greA *mutants, which exhibit the same rank order of effects as in the marker rescue experiment.

**Figure 3:**
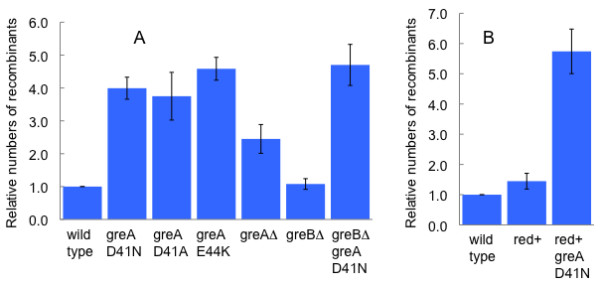
**Transduction**. Strains were infected with an amount of a P1 lysate grown on a *metBΔtet *strain sufficient to generate 50-100 transductants in the wild type. Ratios of mutant transductants to wild type transductants were calculated. Means and standard errors of 3-8 crosses are shown. A. RecBCD+ strains. B. Strains in which *recBCD *is replaced by *red*.

Recombination via the RecA-RecBCD pathway is also affected by *greA *mutations. P1 transduction of the Tn*10 tetRA *genes inserted into several different locations in the chromosome was tested in the D41N mutant. In most, but not all cases, the mutant gave rise to more transductants than the wild type. A marker consisting of the *metB *gene replaced by *tetRA *was typical. As shown in Figure 3, the efficiency of transduction of *metBΔtet *is elevated by all the *greA *mutations. Again, the rank order of effects is the same, though in the opposite direction. The elevated frequency of transduction in the *greA *mutants could be attributed to an elevated frequency of recombination, rather than to a greater efficiency of P1 infection or *tetRA *expression, because P1 transduction of *tetRA *at some other loci, for example *proAB *or *recA*, was not elevated (data not shown).

To characterize further the specificity of *greA *recombination effects, the effect of the *greA*-D41N mutation on RecA-mediated recombination was tested in a strain in which *recBCD *was replaced by *red*. As shown in Figure 3, the replacement has little effect, in either a GreA+ or a *greA*-D41N background. In particular, the *greA *mutation stimulates RecA-Red recombination at least as much as it stimulates RecA-RecBCD recombination.

The GreB protein of *E. coli *is very similar to GreA in structure and biochemical activities [22]. The transcriptional effects of a *greA *deletion mutant are often greater in a strain in which *greB *is deleted as well [23,6,5,8]. However, as shown in Table 1 and Figures 1, 2, and 3, deletion of *greB *had little or no effect on recombination, either by itself or in combination with *greA*-D41N.

The DksA protein of *E. coli*, compared to GreB, is more distantly related to GreA, but it shares a number of structural and mechanistic features with the Gre proteins, including an acid-tipped coiled coil which reaches into the secondary channel of RNA polymerase to regulate transcription [24,25]. Chromosomal *dksA *mutants corresponding to the *greA *mutants described above were constructed and tested for recombination phenotypes: a deletion fusing the first three and last three codons, and a double substitution mutation neutralizing both of the acid fingertip residues, D71N/D74N [24,26]. In contrast to the *greA *mutants, neither *dksA *mutant exhibited either depressed or elevated levels of Red-mediated or RecA-RecBCD-mediated recombination, nor was either resistant to Redβ-SPA (data not shown).

The mechanism by which the *greA *point mutations suppress Redβ-SPA toxicity was investigated by means of a complementation test. Wild type, D41N, and E44K alleles of *greA*, under control of their own promoters, were cloned in multicopy plasmids which are compatible with the Redβ-SPA expressing plasmids. The wild type *greA *plasmid was found to restore Redβ-SPA sensitivity to a chromosomal *greA*-D41N mutant, indicating that the suppression of Redβ-SPA toxicity does not result from a gain of function by the mutant GreA protein. Consistent with this conclusion, and more remarkably, the mutant plasmids do not confer Redβ-SPA resistance upon wild type *E. coli*, indicating that the wild type phenotype prevails even when the mutant gene has a higher copy number. However, interpretation of this latter result is complicated because the mutant plasmids significantly slow the growth of wild type cells, as expected based on the known toxicity of the mutant GreA proteins expressed at high levels [18].

The known biochemical properties of GreA substitution mutants suggest that one effect of the D41N or E44K mutation would be to increase the amount of time RNA polymerase spends stalled in certain backtracked transcriptional complexes. In the mutant, it is likely that other cellular proteins which respond to stalled transcriptional complexes will play a greater role in resolving them. One such protein is Mfd, which not only dissociates stalled transcription complexes, but at the same time recruits proteins that repair DNA lesions [27]. This line of reasoning leads to the question of whether mutant GreA suppression of Redβ-SPA toxicity is mediated by Mfd. Accordingly, wild type and *greA*-D41N strains lacking the *mfd *gene were constructed. Deletion of *mfd *was found to have no effect on either the Redβ-SPA resistance of the D41N mutant, or the Redβ-SPA sensitivity of wild type. It can be concluded that Mfd is not required for *greA*-D41N suppression of Redβ-SPA toxicity.

## Discussion and Conclusions

The experiments described here show that *E. coli greA *mutants exhibit elevated levels of RecA-mediated recombination, and depressed levels of Red-mediated, RecA-independent recombination. GreA is a transcription factor, so these differing effects could be due to changes in gene expression, but recombination and DNA metabolism functions are not among the genes identified as significantly up- or down-regulated in a *greA *null mutant [5]. A more likely explanation comes from the idea that GreA functions to prevent stalling of replication forks that run into conflicts with transcription complexes [7,8].

Replication in *E.coli *is highly processive, and maintains this processivity despite the presence on the template DNA of transcription complexes which have an intrinsic ability to stall replication forks [28]. Stalled replication forks are a potentially dangerous source of genetic instability, because they generate double-strand breaks through a process of RuvABC-mediated reversal and cleavage [29]. The dsDNA ends resulting from these breaks are recombinogenic [30], so loss of a function which promotes the progression of replication forks is predicted to stimulate RecA-RecBCD and RecA-Red recombination events, which proceed via mechanisms that are largely replication-independent [31]. Red-mediated recombination in the absence of RecA, on the other hand, is itself strongly replication-dependent [15,32], so loss of a function which promotes the progression of replication forks might be expected to diminish it.

The *greA *mutant phenotypes reported here are consistent with a model in which replication forks stall either more frequently or for a longer time in a cell in which GreA is inactive. Replication processivity in the mutant then becomes dependent upon other transcription-replication mediators. A functionally defective GreA protein, such as D41N or E44K, which can still bind to its normal site on RNA polymerase, might have an exacerbated effect, by competing with one or more of the other mediators.

Two recent studies present evidence for a role of GreA in promoting the progression of replication forks. (1) Deletion of *greA *increases the UV sensitivity of a *ruvC *mutant. This effect of the *greA *mutant is suppressed by the RNA polymerase open complex-destabilizing *rpoB *mutant H1244Q. The investigators also found that GreA and DksA together contribute to the viability of a *ruvC *strain. They interpreted their observations as indicating that these transcription factors act at DNA lesions to resolve conflicts between DNA replication and transcription [7]. (2) Cultures of an *E. coli dnaC-ts *mutant were synchronized by temperature shift, then, after replication initiation, treated with serine hydroxamate to induce amino acid starvation. The treated cells completed the round of replication they had started, but failed to do so if they lacked DksA, or if they lacked both GreA and GreB. The replication block could be eliminated by additional treatment with rifampicin, or by a DksA-bypassing mutation in *rpoB*, P546L, indicating that RNA polymerase was responsible for the replication block, and that the transcription factors affected replication through their well-characterized interaction with RNA polymerase. The replication deficiency of the *dksA *mutant in this experiment could be complemented by overexpression of GreA from a plasmid. In synchronized *dnaC *mutant cells that were not treated with serine hydroxamate, deletion of *dksA *slightly reduced the rate of replication fork progression; deletion of *dksA*, *greA*, and *greB *all together substantially reduced it. [8]. To these two lines of evidence, a third is now added: in otherwise wild type cells, not treated with DNA damaging agents or metabolic inhibitors, *greA *mutations stimulate replication-independent recombination, and depress replication-dependent recombination.

Stimulation of replication-independent, RecA-RecBCD-mediated recombination was not uniform, but rather was found to vary among different loci. This observation is consistent with the idea that the stimulation is due to the production of dsDNA ends as a byproduct of replication-transcription conflicts: such conflicts would not be expected to occur at a uniform frequency around the chromosome, because different loci are transcribed at different rates.

Alteration of either of GreA's key active site residues has stronger recombination effects than simply eliminating GreA. This observation is consistent with previous studies of many GreA mutants, including D41N and D41A as well as other substitutions for both D41 and E44. These mutants generally reduce or eliminate GreA-induced transcript cleavage, but not binding to RNA polymerase [18-20]. Unlike wild type GreA, the mutant proteins inhibit elongation when used at high concentration in vitro [18]. One unexplained observation of the present study is that the D41A substitution was found to have smaller effects on recombination than the D41N substitution, whereas Laptenko et al. [18] found little if any difference in biochemical activities between these two mutants.

Wang and coworkers propose that the various transcription-replication mediators, including DksA, GreA, GreB and Mfd, could be specialized to prevent transcription-replication conflict under different conditions [8]. The experiments described here provide additional support for this interpretation: based on the recombination and Redβ-SPA sensitivity phenotypes, among these proteins, only GreA appears to have a significant role in mediating transcription-replication conflicts that arise during rapid growth in rich medium. Some qualitative observations on the growth properties of *greA *and *dksA *mutants are also consistent with this idea. The *greA *point mutants grow noticeably more slowly than either wild type or the *greA *deletion on rich medium (LB), but about as well as wild type on minimal medium (M9 glucose-thiamine). In contrast, the *dksA *D71N/D74N mutant grows about as well as wild type on LB, but only a little better on M9 glucose-thiamine than the *dksA *deletion mutant, which, as previously reported, grows very poorly on minimal medium [33].

## Methods

### Strain construction

Redβ was fused to the SPA affinity tag as described by Zeghouf et al. [16], on plasmid pKM208 [34]. The resulting plasmid, pTP1205, contains the *lacI *gene, a synthetic operon in which λ *gam *and *redβ-SPA *are transcribed from *Ptac*, and genes conferring resistance to ampicillin *(bla) *and kanamycin (*neo*). A derivative, pTP1206, was made by deleting *bla*. Further plasmid construction details are given in Table 2. The growth of wild type *E. coli *bearing pTP1205 or pTP1206, but not pKM208, is inhibited by addition of the inducer isopropyl-β-D-thiogalactoside (IPTG). For selection of Redβ-SPA-resistant mutants, the reduced-genome *E. coli *strain MDS12 [35] was mutagenized with *N*-methyl-*N'*-nitro-*N*-nitrosoguanidine plus chloramphenicol [36]. Plasmid pTP1206 was introduced into the mutagenized cells by electroporation. Kanamycin- and IPTG-resistant colonies were selected, and then screened, or re-selected, for the ability to grow while bearing pTP1205 in the presence of IPTG.

**Table 2:** Plasmids

pCP20	[45]. Temperature-sensitive, with heat-inducible FLP
pKM208	[34]. bla-Ptac-gam-bet-exo-lacI, temperature-sensitive mutant pSC101 origin

pJL148	[16]. SPA-neo

pTP1068	Plasmid containing bla-P_32_-aacC. See the description of pTP1232 below.

pTP1205	pKM208 × SP01,02 pcr of pJL148. The Red-mediated recombination event fuses the SPA tag to the C-terminal end of bet, deletes most of exo, and adds neo.

pTP1206	Deletion of bla from pTP1205 by digestion with PvuI and religation

pTP1214	pKM208 × cat29,30 pcr of Tn9 cat. The Red-mediated recombination event replaces a segment of pKM208 between lacI and the replication origin with the cat gene, flanked by BamH1 sites.

pTP1215	Deletion of cat from pTP1214 by digestion with BamH1 and religation

pTP1216	pTP1215 × cat27,28 pcr of Tn9 cat. The Red-mediated recombination event replaces bla and the f1 replication origin with cat. Smaller, chloramphenicol resistance-conferring version of pKM208.

pTP1222	A segment of λ wild type DNA was amplified by SR2,3 pcr. The product was digested with BamH1 and XbaI, and ligated with a BamH1- and XbaI-ended plasmid segment consisting of the Tn903 aph gene and the pSC101 replication origin.

pTP1223	Deletion of sequences between the EcoRV and XbaI sites of pTP1222. The resulting plasmid contains λ sequences from 45262 in the S gene to 45828 in the R gene.

pTP1228, 1230, 1231	Wild type, D41N, and E44K alleles of greA, respectively, amplified by PCR with primers greA5 and greA6, and cloned between the EcoR1 and BamH1 sites of a derivative of pBR322 lacking the sequences between the BamH1 and PvuII sites.

**Figure 4:**
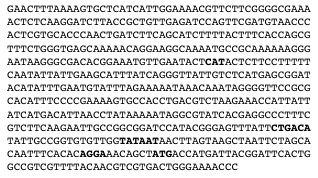
**Sequence of the 587-bp dsDNA used in the tests of Red-mediated chromosomal gene replacement**. The first and last 50 bases are the homology flanks. Bases in bold type are, in order, the start codon of bla (reverse complement), the -35 and -10 hexamers of P_32_, and lacZ ribosome binding site.

pTP1232	Contains the sequences used to construct TP1234, DNA from which in turn was used as the template for PCR synthesis of the 587-bp dsDNA used in the tests of Red-mediated chromosomal gene replacement (sequence shown in Figure 4). It includes the bla gene of pBR322, the synthetic, unregulated promoter P_32 _(closely related to CP32 [46]), the lacZ ribosome binding site, and the N-terminal end of lacZ.

Mapping of the Redβ-SPA-resistant mutants was done as described by Ortenberg et al. [37], except that the mini transposon conferred resistance to tetracycline instead of chloramphenicol. Each mutant was infected with λNK1098 to generate a population with random tetracycline-resistant transposon insertions. Lysates of phage P1 grown on the populations were used to transduce wild type cells to tetracycline- and Redβ-SPA-resistance simultaneously. The mutation-linked transposons were then located by the use of arbitrary PCR and sequencing; oligonucleotides used for this purpose are described in Table 3. The three *greA *mutants were first localized to the vicinity of *yrbD *and *yrbC*. An *argG::cat *insertion was then constructed, and three-factor crosses were employed to determine that the location of the mutations was between *argG *and *yrbD *(91% and 86% co-transduction, respectively). Sequencing of the region revealed that the three mutants had base substitutions altering *greA *codons 41 (GAC to AAC, D41N, isolated twice independently) or 44 (GAA to AAA, E44K), among other, uncorrelated mutations in nearby genes.

**Table 3:** Oligos

aof2	TCCGTAATCATGGTCATAGCTGTTTCCTGTGTGAAATTGTTTAGGTGGCGGTACTTGGGT
ARB1^a^	GGCCACGCGTCGACTAGTACNNNNNNNNNNGATAT

ARB2^a^	GGCCACGCGTCGACTAGTACNNNNNNNNNNACGCC

ARB6^a^	GGCCACGCGTCGACTAGTAC

bpa	TCTGGTGGCCGGAAGGCGAAGCGGCATGCATTTACGTTGAGGTCTGACAGTTACCAATGC

BLZ3	GAACTTTAAAAGTGCTCATCATTG

BLZ4	GGGTTTTCCCAGTCACG

cat27	TCGGAGATCCCCCGGGCTGCAGGAATTCGATATCAAGCTTATGAGACGTTGATCGGCACG

cat28	AGTGGAACGAAAACTCACGTTAAGGGATTTTGGTCATGAGATTCAGGCGTAGCACCAGGC

cat29	GACAGGTTTCCCGACTGGAAAGCGGGCAGTGAGCGCAACGGATCCATGAGACGTTGATCGGCACG

cat30	TGGCTAAATACGGAAGGATCTGAGGTTCTTATGGCTCTTGGATCCATTCAGGCGTAGCACCAGGC

cat34	TCAAAATCGTCAACCCGATTATGGGCGTGAAATTCTGGGAATGAGACGTTGATCGGCACG

cat35	GCGTACTGTGACTTCTTCTGCCGGGATCTTCACGCTCTCAATTCAGGCGTAGCACCAGGC

cat38	AGGTTGATATTGATTCAGAGGTATAAAACGAATGAGTACTATGAGACGTTGATCGGCACG

cat39	CCTACCCGGATATTATCGTGAGGATGCGTCATCGCCATTGATTCAGGCGTAGCACCAGGC

cat40	CACCGTTACACATATGCAGGATGAAGCAGCCAACTTCCCGATGAGACGTTGATCGGCACG

cat41	CGCAGTTCGAGGCTGAACTCTTCTTCCTGGGCTGCACGGTATTCAGGCGTAGCACCAGGC

D41A	AGCTGCGTGGTATTCGGCGTTTTCTTTCAGGGCGCCATGCTCACGCGCTTCCGCGATAGCAGCAA

D41N	AGCTGCGTGGTATTCGGCGTTTTCTTTCAGGTTGCCATGCTCACGCGCTTCCGCGATAGCAGCAA

dksAD	AGTGCGTGTTAAGGAGAAGCAACATGCAAGAAATGGCTGGCTAATTACAGCCGTTCCATCACGT

dksNN	CATATGCAGGATGAAGCAGCCAACTTCCCGAACCCGGTAAACCGTGCAGCCCAGGAAGAAGAGTTCAGCC

E44K	CTGTTCACGAGCTGCGTGGTATTCGGCGTTTTTTTTCAGGTCGCCATGCTCACGCGCTTCCGCGA

greA5	CATCATCATGAATTCAGCTGGTCAATCCGATGA

greA6	ACTACTACTGGATCCTGTACAGGTCATCGGTAAAACG

greA9	TGAAATCATTGCTGCTATCGCGGAAGCGCGTGAGCATGGCCTCGACATCTTGGTTACCGT

greAD	ACATCTTGAGTATTGGGTAATTCTTACAGGTATTCAGCTTGCATTTGTTATACCTCTTGAATATTCCTGA

greAX	CTGTTCACGAGCTGCGTGGTATTCGGCGTTTTCTTTCAGGCGCGGAATAACATCATTTGG

greAY	GCTGCTATCGCGGAAGCGCGTGAGCATGGCGACCTGAAAGCTCGACATCTTGGTTACCGT

greAZ	AGAAACCCTGCTGTTCACGAGCTGCGTGGTATTCGGCGTTCGCGGAATAACATCATTTGG

PRO1	TGCTCATCATTGGAAAACGTTCTTCGGGGCGAAAACTCTACAACCAATTAACCAATTCTG

PRO2	GTCACGACGTTGTAAAACGACGGCCAGTGAATCCGTAATCACGTTGTGTCTCAAAATCTC

Rtet1^a^	CCACTTAATGTGATCATTGAACC

Rtet2^a^	AACCATTTTCAGTGATCCATTGC

SP01	ATTCCTGAAACAGAAAGCCGCAGAGCAGAAGGTGGCAGCATCCATGGAAAAGAGAAG

SP02	TTGCTCCCCAAATACAAAACCAATTTCAGCCAGTGCCTCGCATATGAATATCCTCCTTAG

SR2	GACTACGACTACATCTAGATTCGAACATCGATATGGGCAACTCTATC

SR3	ATCAGCCATCATGGATCCGTTTGCAATGGCGTACCTT

a. Arbitrary PCR and sequencing to locate transposon insertions was done as described [47], but with primers designed for Tn10d(tet). The first round of PCR was done with primers Rtet1, ARB1, and ARB6; the second with Rtet2 and ARB2. Sequencing was done with Rtet2.

Mutations were introduced into the *greA *gene of *E. coli *strain MG1655 by the use of Red-mediated recombination, in a two-step process. The first two bases of codon 41 or 44 were replaced with a tetracycline-resistance cassette. Oligonucleotides were then used to delete the cassette and insert the new bases [9]. Recombinants were identified following kanamycin enrichment for tetracycline-sensitives; details of the protocol are given below. A deletion allele fusing the first three and last three codons of *greA *was constructed the same way. The *greA *alleles were verified by sequencing. The same processes were used to construct chromsomal *dksA*-D71N/D74K and *dksA *deletion mutants, except that the initial construction replaced ten bases in codons 71-74 with the *cat *gene. The *greB *mutation used in some of the experiments was introduced by P1 transduction as *greBΔkan-FRT*; the kanamycin resistance element in it was subsequently excised by the use of FLP recombinase as described [38]. The *mfd *gene was deleted the same way. Other genetic markers were introduced into *greA, greB*, or *dksA *mutant strains by P1 transduction. Further strain construction details are given in Table 4.

**Table 4:** Strains

MG1655	Wild type	[43]
MDS12	Reduced genome version of MG1655	[35]

JW1100	mfdΔkanFRT	[38]

JW3369	greBΔkanFRT	[38]

TP796	MDS12 recAΔtet	[44]

TP798	MG1655 recBCDΔPtac-gam-bet-exo-cat	[44]

Strains constructed for this study. All are in the MG1655 background		

TP933	recBCDΔPtac-gam-bet-exo-cat lacIPΔ::bla-P_32_-aacC-lacZ	TP798 × bpa1,aof2 pcr of pTP1068

TP965	recBCDΔPtac-gam-bet-exo-cat lacIPΔbla'-kan-'lacZ	TP933 × PRO1,2 pcr of Tn903 aph

TP1195	greA::tet41	MG1655/pKM208 × greA9,X pcr of Tn10 tet

TP1198	greA-D41N	TP1195/pKM208 × oligo D41N

TP1201	recAΔtet	MG1655 × P1•TP796

TP1202	greA-D41N recAΔtet	TP1198 × P1•TP796

TP1203	greA::tet44	MG1655/pKM208 × greAY,Z pcr of Tn10

TP1204	greA-D41A	TP1195/pKM208 × oligo D41A

TP1205	greAΔ	TP1195/pKM208 × oligo greAD

TP1208	greA-D41A recAΔtet	TP1204 × P1•TP796

TP1209	greAΔ recAΔtet	TP1205 × P1•TP796

TP1213	greAΔ greBΔtet	TP1205 × P1•TP1199

TP1216	greA-E44K	TP1203/pKM208 × oligo E44K

TP1218	greBΔkanFRT	MG1655 × P1•JW3369

TP1219	greA-E44K recAΔtet	TP1216 × P1•TP796

TP1220	greA-E44K greBΔtet	TP1216 × P1•TP1199

TP1222	greBΔFRT	TP1218/pCP20 passaged at 42°

TP1224	greA-D41N greBΔkanFRT	TP1198 × P1• JW3369

TP1225	lacIPΔbla'-kan-'lacZ	MG1655 × P1•TP965

TP1226	greA-D41N lacIPΔbla'-kan-'lacZ	TP1198 × P1•TP965

TP1227	greA-D41A lacIPΔbla'-kan-'lacZ	TP1204 × P1•TP965

TP1229	greA-E44K lacIPΔbla'-kan-'lacZ	TP1216 × P1•TP965

TP1230	recBCDΔred-cat greA-D41N	TP1198 × P1•TP798

TP1231	greA-D41N greBΔFRT	TP1224/pCP20 passaged at 41°

TP1232	greBΔFRT lacIPΔbla'-kan-'lacZ	TP1222 × P1•TP965

TP1233	greA-D41N greBΔFRT lacIPΔbla'-kan-'lacZ	TP1231 × P1•TP965

TP1234	lacIPΔ::bla-P_32_	TP1225/pTP1216 × pTP1232 linear

TP1235	greBΔFRT recAΔtet	TP1222 × P1•TP796

TP1236	greA-D41N greBΔFRT recAΔtet	TP1231 × P1•TP796

TP1241	greAΔcat lacIPΔbla'-kan-'lacZ	TP1193 × P1•TP965

TP1242	greAΔ lacIPΔbla'-kan-'lacZ	TP1205 × P1•TP965

TP1252	mfdΔkanFRT	MG1655 × P1•JW1100

TP1253	greA-D41N mfdΔkanFRT	TP1198 × P1•JW1100

TP1254	greA-E44K mfdΔkanFRT	TP1216 × P1•JW1100

TP1255	dksAΔ10::cat	MG1655/pKM208 × cat40,41 pcr of Tn9

TP1257	mfdΔFRT	TP1252/pCP20 passaged at 41°

TP1258	greA-D41N mfdΔFRT	TP1253/pCP20 passaged at 41°

TP1259	greA-E44K mfdΔFRT	TP1254/pCP20 passaged at 41°

TP1260	dksA-D71N/D74N	TP1255/pKM208 × oligo dksNN

TP1262	dksA-D71N/D74N recAΔtet	TP1262 × P1•TP796

TP1263	dksAΔ	TP1255/pKM208 × oligo dksAD

TP1264	dksAΔ recAΔtet	TP1263 × P1•TP796

### Kanamycin enrichment for chloramphenicol-sensitive or tetracycline-sensitive variants

Following electroporation, bacteria were diluted into 15 ml of LB, and left standing overnight at 30°C for complete recovery from electroporation and outgrowth of fully sensitive recombinants. 0.2 ml of the culture was diluted into 5 ml LB and aerated at 37°C until a density of about 7 × 10^7^/ml was reached. At that point, 2.5 ml of culture was mixed with 2.5 ml LB plus 100 μl of either chloramphenicol at 1 mg/ml or tetracycline at 0.4 mg/ml, and aerated at 37°C for 40 min. At that time, 2 ml of culture was mixed with 2 ml LB plus 40 μl chloramphenicol or tetracycline, plus 4 μl kanamycin at 20 mg/ml (for a final kanamycin concentration of 20 μg/ml), and aerated at 37°C for 40 min, then left standing overnight at room temperature. Portions of the culture were plated on LB, and the resulting colonies were tested for chloramphenicol or tetracycline sensitivity. Clones bearing the intended deletion were identified by the use of PCR and sequencing.

### Phages

λNK1098 bears the mutations cI857 and P-am80, as well as a mini Tn10 tet element and the Tn10 transposase fused to Ptac. It is similar to λNK1323 [39], but its transposase is wild type, rather than the Altered Target Specificity double mutant version borne by λNK1323. The amber mutation R5 [40], used in marker rescue experiments, was sequenced and found to change λ 45710 from G-C to A-T, which turns R codon 73, UGG, into UAG. The γ210 mutation [21], used in plating tests, was sequenced and found to change λ 33076 from G-C to A-T, which turns *gam *codon 53, CAG, into UAG. The deletion-substitution mutation *redΔcat*, used in plating tests, was constructed by the use of Red-mediated recombination [41], with a dsDNA made by PCR amplification of Tn9 *cat *with oligo primers cat38 and cat39 (Table 3). The deletion-substitution leaves in place the first three codons of *bet *and the last three codons of *exo*.

### Gene-replacement recombination test

Strains to be tested for Red-mediated chromosomal gene replacement were transformed with plasmid pTP1216, a derivative of pKM208 in which *bla *is replaced by *cat*. They were grown at 30°C in LB broth supplemented with chloramphenicol at 20 μg/ml, induced with 1 mM IPTG, and prepared for electroporation as described by Murphy and Campellone [34]. Cells were electroporated with a mixture of 150 ng of a dsDNA generated by PCR of plasmid pTP1232 (Table 2) with oligo primers BLZ3 and BLZ4 (Table 3), plus 1 ng of plasmid pMB9 [42]. The linear dsDNA was 587 bp, including 50 bp homology-targeting flanks (Figure 4). Following electroporation, the cells were diluted into 3 ml LB broth, grown with aeration for 2 hr at 37°C, and plated at 37°C on LB agar supplemented with ampicillin at 100 μg/ml, or tetracycline at 15 μg/ml. To adjust for variable growth rates and electroporation efficiencies, the ratio of ampicillin-resistant (recombinant) to tetracycline-resistant (transformant) colonies, normalized to that of the wild type control, was used as a measure of relative recombination proficiency.
